# Supplementary figures and images for: Metagenomic 16S rDNA reads of in situ preserved samples revealed microbial communities in the Yongle blue hole
Source: PeerJ. 2023 Nov 3;11:e16257. doi: 10.7717/peerj.16257 (PMC10629384; doi:10.7717/peerj.16257)

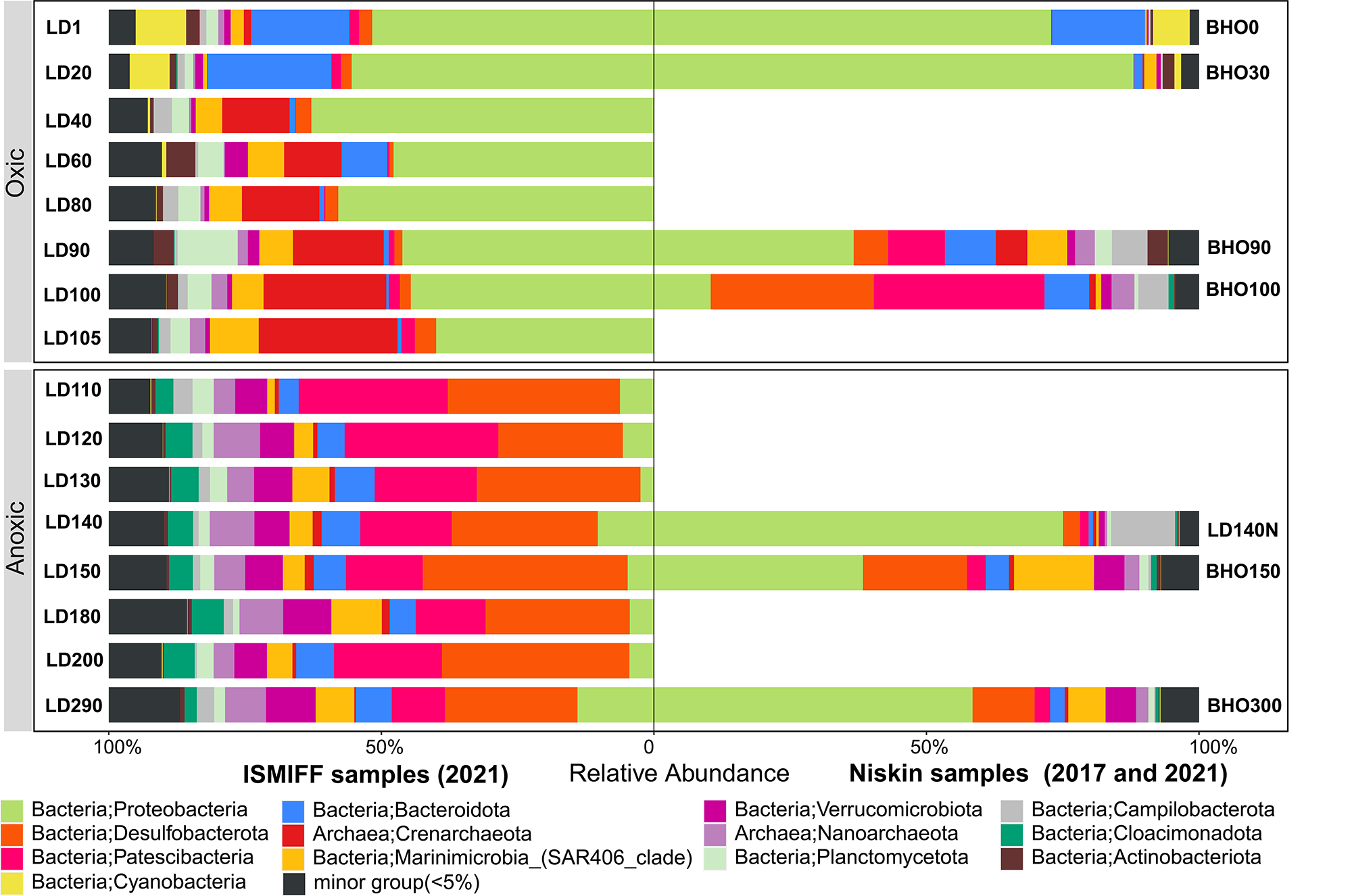

Supplement: Supplemental Information 1 — The community structures were revealed by classification of 16S miTags identified from metagenomes for ISMIFF and Niskin samples of YBH at different depths. The phyla that accounted for less than 5% of the OTUs for all samples were grouped into “minor group”. Sample IDs refer to Tables S1 and S2. Sampling depth is indicated by the number in an ID. LD140N is the only Niskin sample collected in 2021, and the others were collected by He et al. in 2017 (He et al., 2020). [file peerj-11-16257-s001.png]

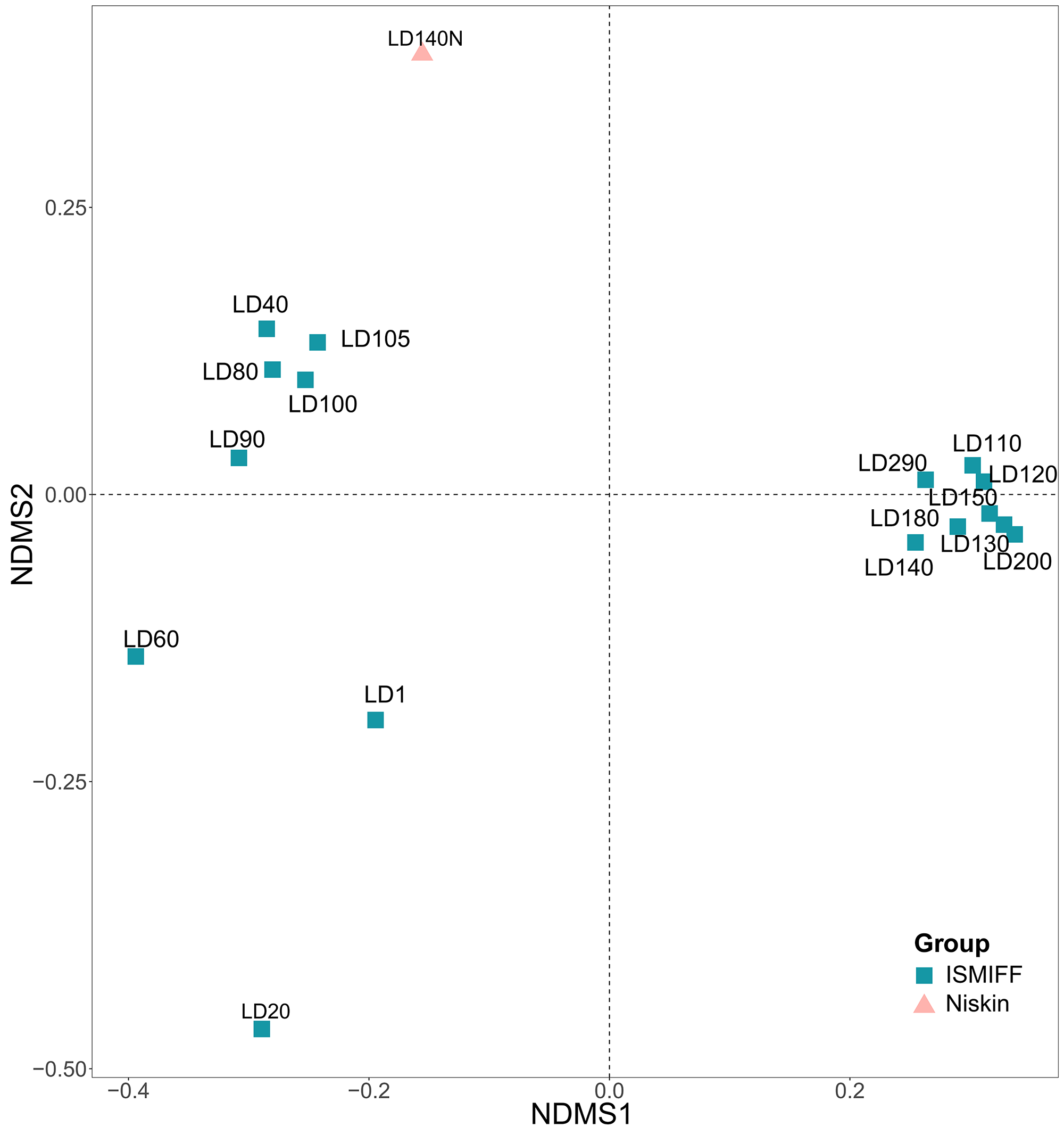

Supplement: Supplemental Information 2 — The relative abundances of orders based on taxonomic classification of 16S miTags were used for the NMDS (stress = 0.04). [file peerj-11-16257-s002.png]

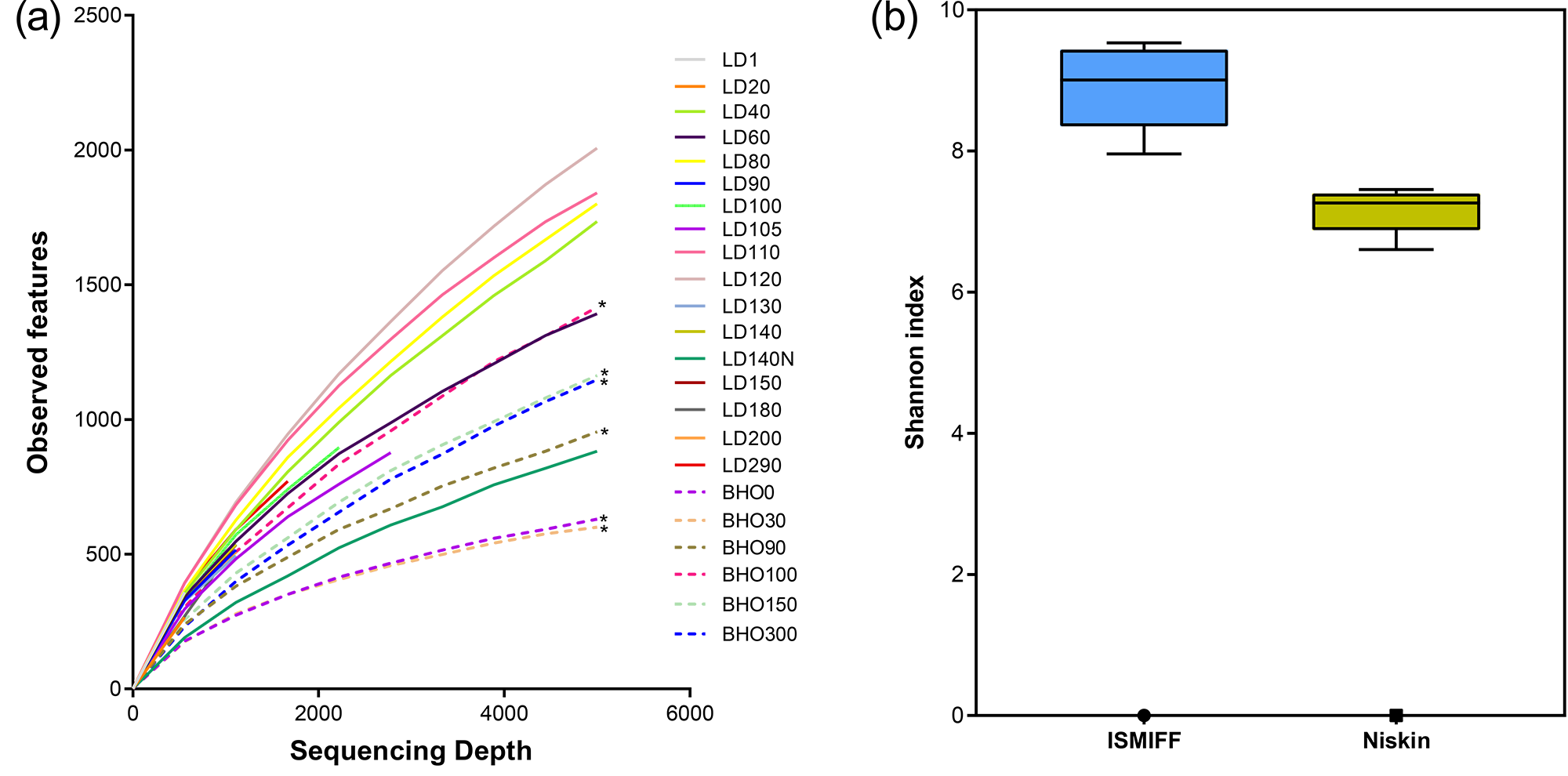

Supplement: Supplemental Information 3 — The OTUs (a) and Shannon index (b) were calculated at a 3% dissimilarity using 16S miTags from V4 region (a). The asterisks indicate the metagenomes for Niskin samples collected in 2017 (He et al., 2020). [file peerj-11-16257-s003.png]
